# Association between Social Integration, Social Exclusion, and Vaccination Behavior among Internal Migrants in China: A Cross-Sectional Study

**DOI:** 10.3390/ijerph19137915

**Published:** 2022-06-28

**Authors:** Jun Wang, Yang Bai, Jingmin Zhu, Xueyao Wang, Yue Che, Jue Liu

**Affiliations:** 1 Center for Health Policy Research and Evaluation, Renmin University of China, Beijing 100872, China; 2018200483@ruc.edu.cn (J.W.); baiyangbeatrice@ruc.edu.cn (Y.B.); 2020000339@ruc.edu.cn (X.W.); 2015311360@email.cufe.edu.cn (Y.C.); 2 School of Public Administration and Policy, Renmin University of China, Beijing 100872, China; 3 Department of Epidemiology and Public Health, University College London, London WC1E 7HB, UK; 4 Department of Epidemiology and Biostatistics, School of Public Health, Peking University Health Science Center, Beijing 100191, China; 5 Institute for Global Health and Development, Peking University, Beijing 100871, China; 6 Key Laboratory of Reproductive Health, National Health Commission of the People’s Republic of China, Beijing 100083, China

**Keywords:** influenza vaccination rates, social integration, social exclusion, migrants

## Abstract

Cross-sectional studies about the association between social integration, social exclusion, and vaccination behavior among internal migrants in China are lacking. In this study, we aimed to explore the association between the influenza vaccination behavior and social integration as well as social exclusion in China based on a cross-sectional study. We included 12,467 participants aged 15 years old or above from the 2017 Migrant Population Dynamic Monitoring Survey (MDMS). We used univariate analysis and logistic regression models to access the association between social integration, exclusion status, and influenza vaccination rates. Results suggested that the association between social integration and the vaccination rate was significantly positive. Moving between different districts impact on people’s mental health and their health performance. Significant association between influenza vaccination behavior and education attainment, income status, health record, and awareness of basic public health services program was reported. Therefore, in order to reduce the incidence of influenza disease and increase the vaccination rate, policymakers and the public should promote social integration for internal migrants. Meanwhile, our finding also implies possible strategies to promote COVID-19 vaccination.

## 1. Introduction

According to the seventh national census of China, the total number of internal migrants in China was 375.82 million in 2020, which increased 69.73% compared with the data of the sixth national census of China in 2010 [1]. Internal migrants (hereinafter as migrants) are defined as individuals who migrate between regions within one country [2,3]. The change in above figures show that great attention should be paid to the growth of migrants. Moreover, some research has also pointed out that the rapid-rising migrant population in China had poor health status, such as their immunization status [4]. As most of Chinese domestic migrants are flowing from rural areas into cities, their living conditions are relatively worse. They are exposed to more health risks, such as the threat of infectious diseases and a lack of planned immunization services. Based on the large proportion of migrants and their severe health conditions, improving the health level of migrants is also significant for achieving the goal of universal health coverage (UHC).

Vaccination was one of the cost effective and efficient methods of preventing infectious diseases [1,5,6,7]. A vaccine is also considered to be a product with a high cost–benefit rate [8]. The Expanded Programme on Immunization (EPI) was promoted globally and also regarded as a very important stage of vaccination in China [6]. In this period, people were required to be vaccinated with some essential vaccines. However, vaccination coverage is still below the Healthy People 2010 and 2020 goals [9]. In particular, it was shown that the coverage levels for some adult vaccinations such as influenza vaccination was low [10]. What is more, compared with other countries, the influenza vaccination coverage in mainland China was extremely low. Previous research showed that the influenza vaccination rate of Chinese people had varied from 2005 to 2017. From the results of a meta-analysis, the vaccination rate had reached the highest point in 2010 at 37.3% but dropped to 7% in 2017 due to people tending to forget the severity of influenza and chose not to become vaccinated, which hindered the prevention and treatment of infectious diseases [11]. Although there is an increasing number of regional governments that have begun to pay, partially or fully, for finance-reimbursed influenza vaccination for selected groups, such as Beijing, Dongli district in Tianjin, Karamay, Shenzhen, and Xinxiang cities [12], most people still need to pay for the influenza vaccine at his/her own expense. People aged over 60 years old or less than 6 years old received much attention [13]. However, there is still a lack of research focusing on the influenza vaccination rate. For migrants, whose registration was different from local residents, it was hard for the vaccine managers to inform them to become vaccinated. Therefore, their willingness to vaccinate voluntarily has become one of the most important factors affecting whether they are vaccinated with the influenza vaccine.

In previous studies, social integration can make people change their health behaviors [14] and has been linked to decreased morbidity and mortality [15]. Compared with local residents, the degree of the social integration of migrants into cities is lower, and the migrants tend to reinforce the perceptions of social exclusion [16,17]. Despite the importance of social integration and social exclusion in affecting migrants’ health behaviors, limited evidence has been provided on the impact of social integration and social exclusion on the vaccination behavior among migrants.

Considering existing literature gap, the aim of our study is to explore the association between social integration, social exclusion, and the influenza vaccination rate among migrants in China based on a cross-sectional study. Three research questions are expected to be answered. The first is whether social integration is positively associated with migrants’ influenza vaccination rate. The second is whether social exclusion is negatively associated with their influenza vaccination rate. The third question is whether migrants’ gender, income, or education attainment affects the association between social integration, social exclusion, and influenza vaccination rate.

The COVID-19 pandemic has had a very serious impact on China and other countries in the world. Although the data used in this research were previously collected by COVID-19, the research on the influenza vaccination of migrants can provide effective support in promoting people’s health behavior during the pandemic.

## 2. Conceptual Framework and Hypotheses

Social integration was regarded as the cohesion of social groups, including the integration of social psychology (or emotion) and social structure (or behavior) [18]. It was also defined as an interactive and multi-dimensional dynamic process growth and development, not a stable state [19,20]. Social capital can help migrant workers obtain resources from social networks that deliver instrumental or emotional support.

When comes to the migrant population, social integration was used to understand and explain their behavior, adaptation, self-identity, and other aspects [3]. The lack of social integration may lead to adverse social or emotional effects such as suicide, depression, and so on [21]. According to social capital theory, migrants can obtain resources from their social networks providing instrumental or emotional support. This is the link between social capital and social integration [22].

Regardubg social exclusion, although there are a large number of studies providing a definition of social exclusion, an agreement of the definition is still lacking. However, all these definitions about social exclusion understand that the social exclusion is not only about material poverty and a lack of material resources, but also being marginalized in society [23,24]. Researchers have divided social exclusion to several dimensions, including impoverishment, labor market exclusion, service exclusion, and exclusion from social relationships [25]. In this study, we mainly focus on the emotionally marginalized migrants and their exclusion of service and social relationships in China.

**Figure 1:**
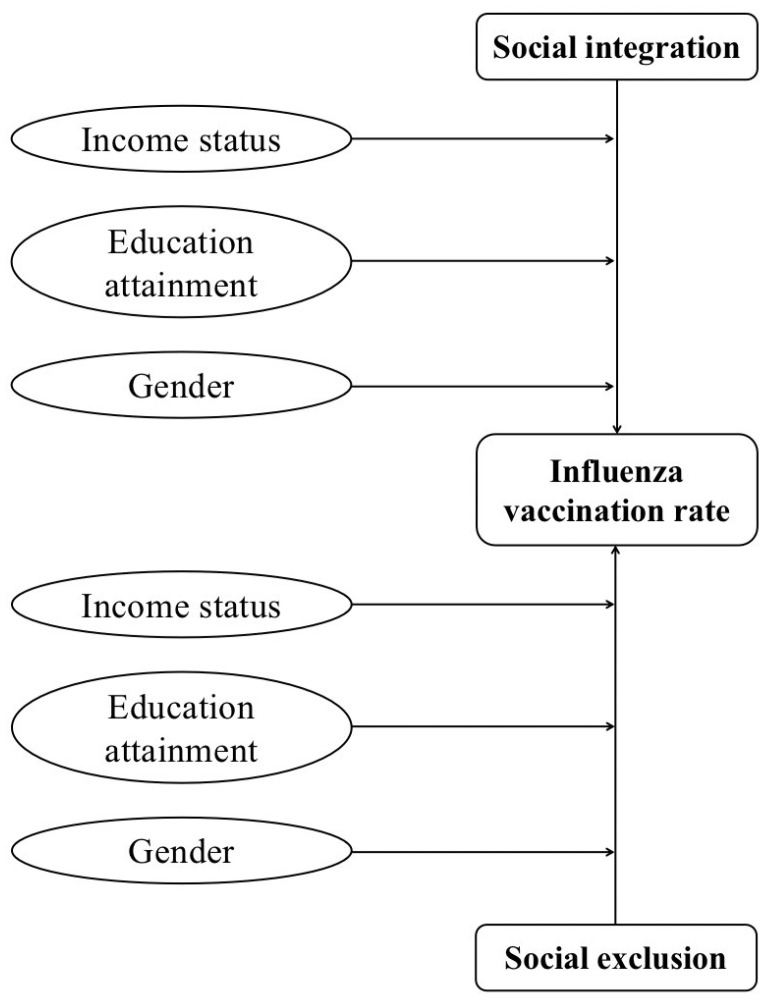
Conceptual framework of this study.

Social integration and exclusion have been essential in determining people’s health behavior [26,27,28]. Studies have shown that social integration can improve people’s health behavior [14]. Meanwhile, social exclusion may lead to adverse social outcomes, especially regarding infectious diseases [29]. What is more, according to Grossman’s health capital theory and health demand model, individual health behavior is affected by demographic factors, socioeconomic factors, environment, individual behavior, and so on [30,31,32]. Therefore, we conducted our conceptual framework as follows (Figure 1).

Based on the theory and literature, the following two hypotheses were constructed.

**Hypothesis** **1.** *Social integration is positively associated with influenza vaccination rate of migrants.*

**Hypothesis** **2.** *Social exclusion is negatively associated with influenza vaccination rate of migrants.*

In fact, social integration is not entirely the opposite of social exclusion. In this study, social integration emphasizes the attention to the city, the love of the city, and the perception of the attitude of the surrounding people towards themselves, which is an integrated indicator. However, social exclusion only emphasizes the bad attitude towards the people around us, which is an indicator of perceived exclusion. That is why we conducted Hypothesis 1 and 2 together in this research.

Previous studies have also pointed out that there were associations between gender, income, education level, and social integration, as well as social exclusion status [33,34,35,36], which may have an impact on a model including these variables and influenza vaccination rate. Regarding gender, research showed that gender may influence the vaccination rates. Men were more likely to become vaccinated while females may care more about the efficacy and safety of the vaccine [36]. Thus, we hypothesize that male migrants are more likely to be affected by social integration and social exclusion, as suggested by the following hypotheses:

**Hypothesis** **3.** *The positive association between social integration and influenza vaccination rate of male migrants is larger in magnitude.*

**Hypothesis** **4.** *The negative association between social exclusion and influenza vaccination rate of male migrants is larger in magnitude.*

It has been shown that pandemic mortality rates were higher among people with the lowest socioeconomic status (SES), implying that low-income and less-educated people needed vaccine protection more than other people [37]. Considering the risk of infectious diseases and the protective effect of vaccination, the poorer and less educated were more likely to be vaccinated against influenza. In this study, we hypothesized that low income migrants are more likely to be affected by social integration and social exclusion. Thus, the following two hypotheses would be tested:

**Hypothesis** **5.** *The positive association between social integration and influenza vaccination rate of low-income migrants is larger in magnitude.*

**Hypothesis** **6.** *The negative association between social exclusion and influenza vaccination rate of low-income migrants is larger in magnitude.*

According to previous studies, people who had higher education attainment would be more likely to be vaccinated [37]. Lacking general knowledge about related issues such as vaccines and the pandemic was also identified as a barrier for vaccination [38]. Hence, we assumed that the vaccination behavior of more-educated people was less likely to be affected by social integration or social exclusion. Hypotheses 7 and 8 were constructed accordingly.

**Hypothesis** **7.** *The positive association between social integration and the influenza vaccination rate of the less-educated migrants is larger in magnitude.*

**Hypothesis** **8.** *The negative association between the social exclusion and influenza vaccination rate of the less-educated migrants is larger in magnitude.*

## 3. Materials and Methods

### 3.1. Sample

Data from the 2017 Migrant Population Dynamic Monitoring Survey (MDMS) were used in this study. This survey was a nationally representative demographic and health survey conducted by the Migrant Population Service Center of the People’s Republic of China (PRC), and the 2017 MDMS survey was conducted from 1 May to 31 May 2017. It used a sampling method called probability proportional to size (PPS), which is a stratified, multi-stage and proportional scale sampling technique [39]. In the process of sampling, the samplers not only pay attention to the scientific nature of the sample, but also consider the actual situation of the sample area.

The 2017 MDMS surveys have two surveys conducted by Migrant Population Service Center of the People’s Republic of China (PRC) at the same time. The first one is the survey on 31 provinces in China and the second one includes a “Key Disease Epidemic Influencing Factors” section, and all basic questions in the first survey is especially conducted in eight provinces, which were Guangdong, Shandong, Jiangsu provinces in the eastern regions, Henan and Hunan provinces and Yunnan in the middle regions, Chongqing, Xinjiang provinces (autonomous region) in the west regions. In the first survey, there are about 170,000 migrants, but the second survey only included 13,998 migrants.

In this research, we use the second survey which included 13,998 respondents and information about vaccination status. Considering that there are great differences in economic development and medical and health levels among eastern, central, and western China, the survey including the “Key Disease Epidemic Influencing Factors” section is conducted in the eastern, central, and western regions of China. The sampling results include three regions in the East, three regions in the middle, and two regions in the west, which are representative to some extent. Moreover, the samples are sampled from the above regions according to the PPS principle, which also shows that the samples are representative.

In this research, the investigators use a “multi-stage” method, in which the sampling is divided into three stages. The first stage is to select townships (towns and streets) by PPS method. The second stage is to select village (neighborhood) committees in the selected townships (towns, streets) according to the PPS method. The third stage is to select individual survey objects from the village (neighborhood) committee. In the aspect of “proportional scale sampling”, the sample allocation proportion between each layer is inconsistent with its proportion in the population. Therefore, it is necessary to give weights to each layer to make the final sample proportion consistent with the population proportion, so as to achieve the purpose of proportional sampling.

Participants in this survey were the migrants who lived in one destination city for more than one month. Each village and neighborhood committee in the sample shall, according to the survey group determined by the state for each sample point, check all the floating population (the sample points of scattered residence types shall be reported to all the current members of 50 migrants’ households) in their households and fill them in on the national sampling platform. In case the respondents cannot be visited during the formal survey, the investigators of each sample point can apply on the national sampling platform, and the country will replace them according to the principle of “the same sex, the same age, and the same residence time”. In principle, the replacement personnel shall be found in the report roster. If there is no suitable replacement object, it shall be expanded to the scope of the village (neighborhood) committee. All of the above information was from the technical documents of 2017 migration population dynamic monitoring survey (MDMS), which needs to be downloaded from the online website in PDF version for reading (https://www.ncmi.cn/phda/dataDetails.do?id=CSTR:A0006.11.A000T.201906.000225) (accessed on 6 June 2019).

**Figure 2:**
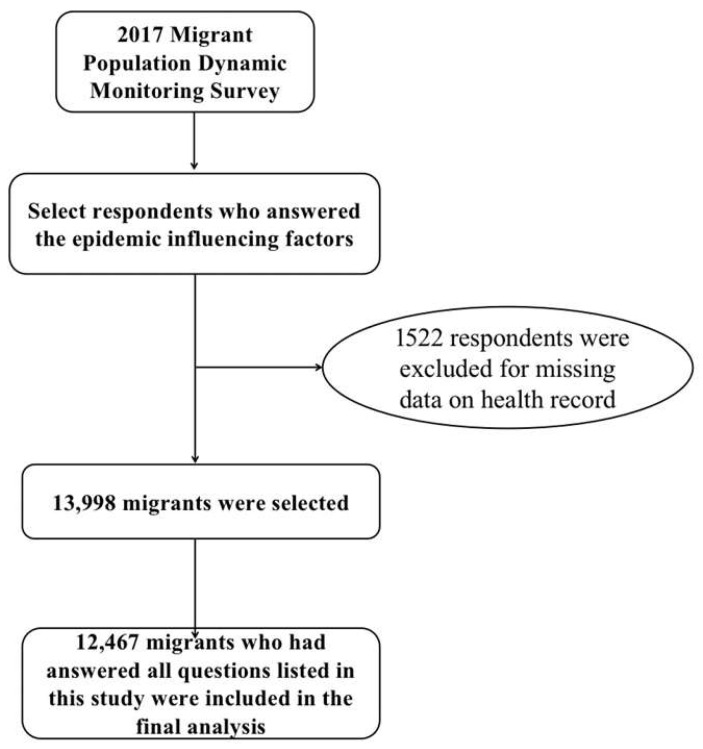
The flow chart of respondents.

At first, we included 13,998 migrants in the initial scope. Then, we counted the answers of each variable and removed the respondents who did not answer the question “Have you established the health record”. The descriptions of dropped samples have shown similar demographic and socioeconomic characteristics regarding gender, age group, marital status, region, and education attainment. Removing these samples did not influence our results in this research. Finally, we obtained 12,467 samples for analysis (Figure 2).

### 3.2. Measures

In order to conduct this research, the interviewers were trained before the investigation, and the investigators included the investigation instructors and investigators for better management. This research was conducted through mobile phones or pads provided by the Investigation Center, which could help the investigators and their instructors follow up the sampling process and review the interview recordings. This could greatly improve the efficiency and accuracy of the survey. China had 829 million Internet users in 2018, accounting for nearly 60% of the total population [40]. Moreover, according to the 49th statistical report on the development of China’s Internet Network released by China Internet Network Information Center (CNNIC) in Beijing, by December 2021, the number of Chinese Internet users had reached 1.032 billion, and the Internet penetration rate had reached 73.0%. According to a smartphone addiction survey including 33,831 participants of 24 countries among the world, the smartphone usage score has the highest point [41]. This has also given support to the survey.

#### 3.2.1. Influenza Vaccination Rate

In this study, we focused on the vaccination of the influenza vaccine, which was essential for people’s daily life. In this survey, the influenza vaccination status was measured by the question “Have you ever been vaccinated the influenza vaccine?” and the answer was yes or no. The influenza vaccination rate was calculated by dividing the number of people who were vaccinated by the total number of people and then multiplying the number by 100%. Our research was aimed at the association between influenza vaccination status and social integration and social exclusion in general, rather than a specific year, such as 2017.

#### 3.2.2. Social Integration

In our study, social integration was also the subjective social integration status in general. It was measured by three questions: “I like the city/place where I live now”, “I pay attention to the changes of the city/place where I live now”, and “I am willing to integrate into the local people and become one of them”. The answers were “totally disagree”, “disagree”, “basically agree”, and “fully agree”, scored 1, 2, 3, and 4, respectively. Finally, it is summed up to form a total score of the social integration of migrants with a value range of 3–12 [42].

#### 3.2.3. Social Exclusion

Social exclusion is also the subjective social exclusion status in general. It was measured by two questions: “I think local people are willing to accept me as one of them” and “I feel that local people always look down on outsiders”. The answers were “completely disagree”, “disagree”, “agree”, and “completely agree”, scored 4, 3, 2, and 1 points to the previous question and 1, 2, 3, and 4 points to the latter question, respectively. The scores were summed up with a value of 2–8 points. The higher the score, the higher the perceived social exclusion [42].

#### 3.2.4. Demographic Characteristics

According to previous studies, there were four demographic characteristics as independent variables in this study: gender (male/female); age (≤30, 31–40, 41–50, >50); marital status (single, including divorced or widowed/married, including having a relationship); and region (rural or urban) [43].

#### 3.2.5. Socioeconomic Characteristics

We included education attainment, health security card, and income status to measure the socioeconomic characteristics. We divided the education attainment into four groups: middle school or below, high school, three-year technical college, university or above. The social security card, which could help citizens save part of their medical insurance expenses, was measured by yes or no.

Income was highly associated with health situation, and income inequality can lead to huge differences in health [44]. However, because this survey did not contain the income variable, we classified the origins of migrants into three regions: the east, the middle, and the west. According to the socioeconomic development level of these three regions, they were divided into three grades: high, middle, and low, which can be used to estimate the income status of migrants.

#### 3.2.6. Health Characteristics

The characteristics about health may also influence the influenza vaccination rate. In this study, we used the five variables to measure the general health status (healthy or unhealthy); hypertension (yes or no); Type 2 diabetes mellitus (T2DM) (yes or no); the possession of an established health record (yes or no); and having knowledge of the basic public health services program (yes or no).

### 3.3. Statistical Analysis

All statistical analyses were performed using Stata15.0 (StataCorpLP., College Station, TX, USA). We used descriptive analysis and logistic regression models to test. Firstly, we used descriptive analysis to figure out the frequency and proportion of each type of characteristics. Meanwhile, the Chi-squared tests were performed to examine the associations between social integration, social exclusion, related characteristics (including demographic, socioeconomic, and health characteristics) and the influenza vaccination rate.

Univariate and multivariate logistic regressions were applied to identify the social integration, social exclusion, and influenza vaccination rate, from which odds ratios (OR) and 95% confidence intervals (95% CI) were calculated. In this process, the interactions were added to the models to analyze the influence made by gender, income, and education attainment to the association. A *p*-value of less than 0.05 (two-tailed) was considered to be a statistical significance in all analyses.

## 4. Results

### 4.1. Basic Characteristics of the Respondents

Characteristics of the 12,467 participants at baseline were shown in Table 1. According to the Chi-squared tests, this table showed that there was great significance in age and marital status in demographic characteristics, education attainment, and income status in socioeconomic characteristics, as well as in hypertension status and in the awareness of the basic public health services program (*p* < 0.001).

The percentage of males was slightly higher than females, and migrants whose age were less than 31 years old took up the largest part. Respondents who were married (including having a relationship) and came from rural places accounted for 81.43% and 82.89%, respectively. In this survey, only 6.46% migrants went to university. The percentages of individuals who have or do not have a social security card were almost the same, and this variable could also significantly relate to the influenza vaccination. Regarding the characteristics about health, healthy people took up almost 98%, and people who suffered from hypertension or diabetes were 4.19% and 0.99%, respectively. The health record and basic public health services program were set to improve people’s health status. From the results, we could see that the number of migrants had already established that the health record was 3925 (31.46%), while the number of those who had been aware of the basic public health services program was 7961 (63.81%).

### 4.2. The Association between Social Integration, Social Exclusion, and Influenza Vaccination Rate

Three logistic regression models were conducted in this study to calculate the associations between social integration, social exclusion, and the influenza vaccination rate. The interactions (including gender * social integration, gender * social exclusion, income * social integration, income * social exclusion, education attainment * social integration and education attainment * social exclusion) were respectively added in models to examine the interaction impact of gender, income, and education attainment (Table 2).

#### 4.2.1. Social Integration and Influenza Vaccination Rate

In model 1 (Table 2), we included social integration, the demographic, socioeconomic, and health characteristics of migrants, as well as interactions related to social integration. The result showed that the influenza vaccination rate was associated with social integration (OR = 1.106; 95% CI: 1.02–1.20). As to demographic characteristics, ages between 41 and 50 years old (OR = 0.611; 95% CI: 0.51–0.73), and above 50 years old (OR = 0.550; 95% CI: 0.42–0.72), were significantly associated with the vaccination rate. In the socioeconomic characteristics section, social security was attributed to the significance of this model (OR = 1.125; 95% CI: 1.00, 1.26). Health characteristics and the awareness of basic public health services program (OR = 1.200; 95% CI: 1.05–1.10) were both associated with influenza vaccination.

In model 3, we put variables about social integration and social exclusion together, and the result also showed that there was a significant relationship between social integration and influenza vaccination rate (OR = 1.142; 95% CI: 1.04, 1.22). When we added social exclusion in model 3, the odds ratio increased. We supposed that, model 3, which included all variables, would be more suitable, comprehensive, and objective.

#### 4.2.2. Social Exclusion and Influenza Vaccination Rate

Different from what we mentioned in Hypothesis 2, there was no association between social exclusion and influenza vaccination rate in all models (Table 2). We included social exclusion and characteristics as well as interactions in model 2 (OR = 1.011; 95% CI: 0.90–1.13), but we could not find any significant association between social exclusion and influenza vaccination rate.

In model 3, while we put all variables including social integration and all interactions, there was still no significance between social exclusion and influenza vaccination rate (OR = 1.062; 95%: 1.00–1.13). Therefore, we thought that Hypothesis 2 was not tenable.

#### 4.2.3. Interactions and Influenza Vaccination Rate

In model 1, high income status showed a negative impact on the relationship between social integration and the influenza vaccination rate (OR = 0.899; 95% CI: 0.83, 0.98). High school education attainment attributed to the relationship between the integration and vaccination rate (OR = 1.093; 95% CI: 1.00, 1.19), while other interactions did not show any significance.

We found that, in model 2, there was no significance in any interactions. In model 3, the significance differed from model 1 and model 2. Considering the results are more robust in model 3, we finally use model 3 to analyze the interaction effects on the influenza vaccination rate. The gender character contributed to the relationship between the social integration and influenza vaccination rate (OR = 0.913; 95% CI: 0.84, 0.99) while not in social exclusion.

## 5. Discussion

**Figure 3:**
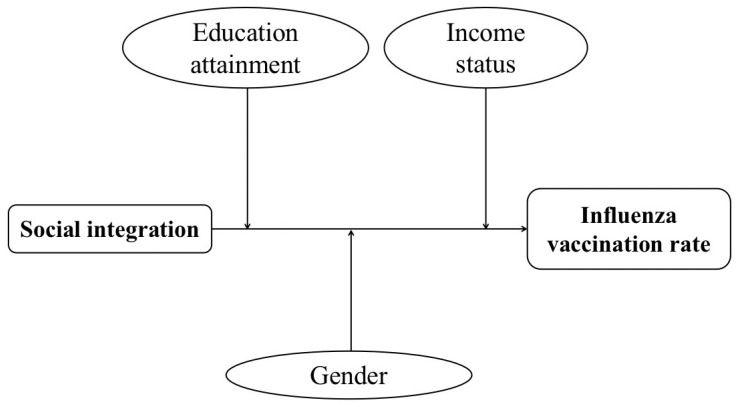
Adjusted framework of this study.

To the best of our knowledge, this is the first study to examine the association between social integration, social exclusion, and influenza vaccination rate of migrants in China. According to our empirical results, we modified our framework (Figure 3). The results indicated that there was significant association between social integration and the influenza vaccination rate of migrants, and the relationship was positive. However, we could not find any association between social exclusion and the influenza vaccination rate.

Our findings on the association between social integration and health behaviors is consistent with the literature [28,38,45]. Social integration was seen as an aspect of social support, and it could positively influence people’s health behavior [45]. Poor social relations might contribute to adverse health behavior [28]. Psychological determinants were related to vaccination uptake [38].

The association between social integration and vaccination behavior might be explained by three reasons: First, individuals are hesitant about vaccination due to psychological factors [46]. Second, social integration may enhance peer effect [47]. If the friends or acquaintances of a migrant were vaccinated or knew more about the vaccines, it can increase the probability of migrant’s vaccination rate. Finally, social integration usually indicates more contact with the local community health service center, which promotes migrants’ perception of the risk of infectious diseases and a higher probability to become vaccinated is incurred [47].

Consistent with previous studies [47,48], our study has also shown the impact of demographic characteristics such as gender and education on vaccination rate. In addition, the association between social integration and vaccination behavior was significantly larger in female migrants. We supposed that women are more vulnerable to this effect due to their stronger perception of social integration than men [49,50]. As to income, people with higher income showed less probability of vaccination associated with social integration, which was plausible, considering possible more worries about vaccination risks in the high-income population. In educational level, the association was larger in migrants with lower educational level, contrary to some existing evidence [37,38]. This may also come down to less educated individuals being less worried about the risk of vaccines.

Studies showed that the vaccination program had significant social value [51]. Social mobilization, communication, and other methods were used to improve the vaccination rate [52]. Based on these studies, we believe that it is extremely significant for policymakers to take measures to enhance the social integration situation of migrants. For instance, the government could provide more public facilities and services for the migrants, such as disease prevention publicity, fitness facilities, counseling centers, etc., in order to promote their integration into the local community.

Different from other research, we not only found the social support impact on health behavior and health decision [28,38], but also figured out the great association between social integration and vaccination behavior, which contributed to a previous study.

Our study also had several limitations. First, because this was a second-hand study, some important information, such as the income, the level of the local healthcare institutions, parental information, and so on, could not be included in our models. Existing research showed that the migrant status of parents would have a greater impact on children’s vaccination. However, the MDMS data based on this article did not contain the relevant situation of the parents, and we could not include this as another control variable [52]. Moreover, other determinants of vaccination behavior, including past experience, perceived risks, media, and so on, were also not included in this study [52]. Finally, we could only estimate the economic situation of the respondents through the inflow provinces, rather than the salary or income of migrants, which might deviate from the actual situation.

## 6. Conclusions

In summary, this study examined the association between social integration, social exclusion, and vaccination behavior among migrants in China. Our findings highlighted the significance for governments to improve social integration for migrants and provide more supportive policies.

Recently, the COVID-19 pandemic might also affect the influenza vaccination rate among migrants. During this special period, their social integration and exclusion status might change, calling for more in-depth research. This study used cross-sectional data collected in 2017. It was not possible to evaluate the impact of COVID-19 pandemic on influenza vaccination. However, due to the similarity between the influenza vaccine and the COVID-19 vaccine for citizens, the results of improving the vaccination rate of the influenza vaccine through social integration can effectively provide reference for the prevention and control of the COVID-19 pandemic.

In the future, we should give migrants better social support for influenza vaccination and provide better protection for these vulnerable groups, especially for the migrants working in the medical and health field [53]. Since social integration plays a great importance in vaccination promotion, improving the social integration level is helpful and beneficial to health service utilization. In the future, we plan to measure the impact of the COVID-19 pandemic on the vaccination, social integration, and social exclusion of migrants through cohort data, and put forward more valuable policy suggestions for the health of migrants under the pandemic.

## Figures and Tables

**Table 1:** Characteristics and influenza vaccination rate of the respondents and univariate analysis at the baseline.

Characteristics	N (%)	Got Vaccinated			
		N (%)	OR	95% CI	*p*-Value
**Total**	12,476 (100)	1414 (11.33)			
**Demographic characteristics**					
**Gender**			1.018	0.91–1.14	0.753
Male	6393 (51.24)	719			
Female	6083 (48.76)	695			
**Age**			0.673	0.60–0.75	0.000 ***
≤30	4627 (37.09)	644			
31–40	4090 (32.78)	473			
41–50	2727 (21.86)	219			
>50	1032 (8.27)	78			
**Marital status**			0.703	0.62–0.80	0.000 ***
Single	2317 (18.57)	335			
Married	10,159 (81.43)	1079			
**Region**			1.028	0.89–1.19	0.707
Rural	10,341 (82.89)	1167			
Urban	2135 (17.11)	247			
**Socioeconomic characteristics**					
**Education attainment**			1.5445	1.38–1.73	0.000 ***
Middle school or below	6941 (55.63)	651			
High school	3235 (25.93)	446			
Three-year technical college	1494 (11.97)	205			
University or above	806 (6.46)	112			
**Social security card**			1.208	1.08–1.35	0.001 **
Have	6433 (51.56)	788			
Do not have	6043 (48.44)	626			
**Income status**			0.725	0.65–0.81	0.000 ***
High income	7166 (57.44)	683			
**Characteristics**	**N (%)**	**Got vaccinated**			
		**N (%)**	**OR**	**95%CI**	***p*** **-Value**
Middle income	1684 (13.50)	233			
Low income	3636 (29.06)	498			
**Characteristics about health**					
**Health status**			1.414	0.90–2.22	0.1134
Healthy	12,224 (97.98)	1393			
Unhealthy	252 (2.02)	21			
**Hypertension**			0.498	0.35–0.72	0.000 ***
Yes	523 (4.19)	32			
No	11,953 (95.81)	1382			
**Type 2 diabetes mellitus(T2DM)** Yes	124 (0.99)	9	0.610	0.31–1.20	0.127
No	12,352 (99.01)	1045			
**Establishment of health record** Yes	3925 (31.46)	512	1.272	1.13–1.43	0.000 ***
No	8551(68.54)	902			
**The awareness of basic public health services program**			1.360	1.206–1.533	0.000 ***
Yes	7961 (63.81)	988			
No	4515 (36.19)	426			

** *p* < 0.01, *** *p* < 0.001.

**Table 2:** Association of social integration, social exclusion, and influenza vaccination of migrants in the multivariable models.

	Model 1	Model 2	Model 3
	OR (95% CI)	OR (95% CI)	OR (95% CI)
**Social integration**	1.106 ** (1.02, 1.20)		1.142 ** (1.04, 1.22)
**Social exclusion**		1.011 (0.90, 1.13)	1.062 (1.00, 1.13)
**Demographic characteristics**			
**Gender (Female)**			
Male	2.046 (0.96, 4.37)	1.056 (0.71, 1.57)	3.408 * (0.95, 4.35)
**Age (≤30)**			
31–40	0.881 (0.77, 1.01)	0.884 (0.77, 1.02)	0.880 (0.76, 1.01)
41–50	0.611 *** (0.51, 0.73)	0.610 *** (0.51, 0.73)	0.609 *** (0.51, 0.73)
>50	0.550 *** (0.42, 0.72)	0.555 *** (0.42, 0.73)	0.550 *** (0.42, 0.72)
**Marital status (Single)**			
Married	0.871 (0.75, 1.01)	0.873 (0.75, 1.01)	0.868 (0.75, 1.01)
**Region (Rural)**			
Urban	0.909 (0.78, 1.06)	0.924 (0.79, 1.08)	0.914 (0.78, 1.07)
**Socioeconomic characteristics**			
**Education attainment (middle school or below)**			
High school	0.563 (0.23, 1.37)	2.030 ** (1.26, 3.26)	0.561 (0.23, 1.37)
Three-year technical college	1.230 (0.38, 3.94)	1.478 (0.80, 2.73)	1.214 (0.38, 3.88)
University or above	0.664 (0.14, 3.23)	1.565 (0.71, 3.44)	0.663 (0.14, 3.24)
**Social security card (do not have)**			
have	1.125 * (1.00, 1.26)	1.130 * (1.01, 1.27)	1.129 * (1.00, 1.27)
**Income status (low)**			
Middle	1.438 (0.42, 4.92)	0.890 (0.46, 1.71)	1.398 (0.41, 4.79)
High	1.869 (0.79, 4.44)	0.426 *** (0.28. 0.66)	1.797 (0.75, 4.28)
**Characteristics about health**			
**Health status (unhealthy)**			
Healthy	1.023 (0.64, 1.64)	1.025 (0.64, 1.64)	1.034 (0.65, 1.65)
Hypertension	0.683 (0.47, 1.00)	0.674 * (0.46, 0.99)	0.680 * (0.46, 0.99)
T2DM	0.909 (0.45, 1.84)	0.919 (0.45, 1.86)	0.913 (0.45, 1.84)
Health record	1.106 (0.97, 1.37)	1.121 (0.99, 1.27)	1.112 (0.98, 1.26)
The awareness of basic public health services	1.200 ** (1.05, 1.01)	1.219 ** (1.09, 1.39)	1.208 ** (1.06, 1.38)
**Interactions**			
Gender * Social integration	0.936 (0.87, 1.01)		0.913 * (0.84, 0.99)
Gender * Social exclusion		0.996 (0.90, 1.11)	0.932 (0.83, 1.05)
Income status * Social integration (low income)			
Middle income	0.962 (0.85, 1.09)		0.954 (0.83, 1.10)
High income	0.899 * (0.83, 0.98)		0.911 (0.93, 1.00)
Income status * Social exclusion (low income)			
Middle income		1.008 (0.85, 1.19)	0.976 (0.80, 1.18)
High income		1.104 (0.98, 1.24)	1.033 (0.90, 1.18)
Education attainment * Social integration			
High school	1.093 * (1.00, 1.19)		1.078 (0.98, 1.19)
Three-year technical college	1.003 (0.90, 1.12)		0.989 (0.86, 1.13)
University or above	1.063 (0.92, 1.24)		1.063 (0.89, 1.27)
Education attainment * Social exclusion			
High school		0.903 (0.80, 1.02)	0.962 (0.83, 1.11)
Three-year technical college		0.962 (0.82, 1.13)	0.957 (0.78, 1.17)
University or above		0.945 (0.76, 1.17)	0.996 (0.77, 1.28)
P for the model	0.000 ***	0.000 ***	0.000 ***

* *p* < 0.05, ** *p* < 0.01, *** *p* < 0.001.

## Data Availability

The availability of these data is limited. The data used in this paper is the 2017 China floating population health and family planning dynamic monitoring health data. The data are from the floating population service center of the National Health Commission of the people’s Republic of China and can be obtained from the data warehouse of the national population health science data center (https://www.ncmi.cn/phda/dataDetails.do?id=CSTR:A0006.11.A000T.201906.000225) (accessed on 6 June 2019).

## References

[1] Wang J., Zhu J., Wang X., Che Y., Bai Y., Liu J. (2021). Sociodemographic disparities in the establishment of health records among 0.5 million migrants from 2014 to 2017 in China: A nationwide cross-sectional study. Int. J. Equity Health.

[2] Lin Y., Zhang Q., Chen W., Shi J., Han S., Song X., Xu Y., Ling L. (2016). Association between social integration and health among internal migrants in ZhongShan, China. PLoS ONE.

[3] Lin Y., Zhang Q., Chen W., Ling L. (2017). The social income inequality, social integration and health status of internal migrants in China. Int. J. Equity Health.

[4] Fang H., Yang L., Zhang H., Li C., Wen L., Sun L., Hanson K., Meng Q. (2017). Strengthening health system to improve immunization for migrants in China. Int. J. Equity Health.

[5] Lee P.Y., Matchar D.B., Clements D.A., Huber J., Hamilton J.D., Peterson E.D. (2002). Economic analysis of influenza vaccination and antiviral treatment for healthy working adults. Ann. Intern. Med..

[6] Streefland P.H. (2001). Public doubts about vaccination safety and resistance against vaccination. Health Policy.

[7] Tsutsui Y., Benzion U., Shahrabani S., Din G.Y. (2010). A policy to promote influenza vaccination: A behavioral economic approach. Health Policy.

[8] Le P., Rothberg M.B. (2018). Limited Focus in Evaluation of Vaccine Cost-effectiveness-Reply. JAMA Int. Med..

[9] Stockwell M.S., Fiks A.G. (2013). Utilizing health information technology to improve vaccine communication and coverage. Hum. Vaccines Immunother..

[10] Leidner A.J., Murthy N., Chesson H.W., Biggerstaff M., Stoecker C., Harris A.M., Acosta A., Dooling K., Bridges C.B. (2019). Cost-effectiveness of adult vaccinations: A systematic review. Vaccine.

[11] Wang Q., Yue N., Zheng M., Wang D., Duan C., Yu X., Zhang X., Bao C., Jin H. (2018). Influenza vaccination coverage of population and the factors influencing influenza vaccination in mainland China: A meta-analysis. Vaccine.

[12] Yang J., Atkins K.E., Feng L., Pang M., Zheng Y., Liu X., Cowling B.J., Yu H. (2016). Seasonal influenza vaccination in China: Landscape of diverse regional reimbursement policy, and budget impact analysis. Vaccine.

[13] Yang J., Atkins K.E., Feng L., Baguelin M., Wu P., Yan H., Lau E., Wu J.T.K., Liu Y., Cowling B.J. (2020). Cost-effectiveness of introducing national seasonal influenza vaccination for adults aged 60 years and above in mainland China: A modelling analysis. BMC Med..

[14] McQuestion M.J., Quijano Calle A., Drasbek C., Harkins T., Sagastume L.J. (2010). Social integration and health behavioral change in San Luis, Honduras. Health Educ. Behav..

[15] Uchino B.N., Landvatter J., Zee K., Bolger N. (2020). Social Support and Antibody Responses to Vaccination: A Meta-Analysis. Ann. Behav. Med..

[16] Liu L., Huang Y., Zhang W. (2017). Residential segregation and perceptions of social integration in Shanghai, China. Urban. Stud..

[17] Qin L., Chen C.P., Wang W., Chen H. (2021). How migrants get integrated in urban China-The impact of health insurance. Soc. Sci. Med..

[18] O’Reilly C.A., Caldwell D.F., Barnett W.P. (1989). Work Group Demography, Social Integration, and Turnover. Adm. Sci. Q..

[19] Zhou J., Zhou J., Zhang H., Zhang J. (2022). Social Integration as Mediator and Age as Moderator in Social Capital Affecting Mental Health of Internal Migrant Workers: A Multi-Group Structural Equation Modeling Approach. Front. Public Health.

[20] Ware N.C., Hopper K., Tugenberg T., Dickey B., Fisher D. (2007). Connectedness and citizenship: Redefining social integration. Psychiatr. Serv..

[21] Berkman L.F., Glass T., Brissette I., Seeman T.E. (2000). From social integration to health: Durkheim in the new millennium. Soc. Sci. Med..

[22] Li T.C., Chu C.C., Meng F.C., Li Q., Mo D., Li B., Tsai S.-B. (2018). Will Happiness Improve the Psychological Integration of Migrant Workers?. Int. J. Environ. Res. Public Health.

[23] Apospori E., Millar J. (2003). The Dynamics of Social Exclusion in Europe.

[24] Atkinson A.B., Atkinson A.B., Hills J. (1998). Social exclusion, poverty and unemployment. Exclusion, Employment and Opportunity.

[25] Pantazis C., Gordon D., Levitas R. (2006). Poverty and Social Exclusion in Britain. Bristol Policy Press.

[26] Feng Z., Jones K., Phillips D.R. (2019). Social exclusion, self-rated health and depression among older people in China: Evidence from a national survey of older persons. Arch. Gerontol. Geriatr..

[27] Sacker A., Ross A., MacLeod C.A., Netuveli G., Windle G. (2017). Health and social exclusion in older age: Evidence from Understanding Society, the UK household longitudinal study. J. Epidemiol. Commun. Health.

[28] Weyers S., Dragano N., Möbus S., Beck E.M., Stang A., Möhlenkamp S., Jöckel K.H., Erbel R., Siegrist J. (2010). Poor social relations and adverse health behaviour: Stronger associations in low socioeconomic groups?. Int. J. Public Health.

[29] He J., He L., Zhou W., Nie X., He M. (2020). Discrimination and Social Exclusion in the Outbreak of COVID-19. Int. J. Environ. Res. Public Health.

[30] Grossman M. (1972). The Demand for Health-A Theoretical and Empirical Investigation.

[31] Grossman M. (1972). On the Concept of Health Capital and the Demand for Health. J. Political Econ..

[32] Grossman M. (2000). The Human Capital Model. Handbook of Health Economics.

[33] Toyama M., Fuller H.R., Owino J. (2022). Longitudinal Implications of Social Integration for Age and Gender Differences in Late-Life Physical Functioning. Int. J. Aging Hum. Dev..

[34] Dahlberg L., McKee K.J., Fritzell J., Heap J., Lennartsson C. (2020). Trends and gender associations in social exclusion in older adults in Sweden over two decades. Arch. Gerontol. Geriatr..

[35] Jo E.B., Kwon R.H., Jung M. (2020). Contextual effects of social integration and disintegration on health status: Evidence from South Korea. BMC Public Health.

[36] Bish A., Yardley L., Nicoll A., Michie S. (2011). Factors associated with uptake of vaccination against pandemic influenza: A systematic review. Vaccine.

[37] Mamelund S.E., Shelley-Egan C., Rogeberg O. (2019). The association between socioeconomic status and pandemic influenza: Protocol for a systematic review and meta-analysis. Syst. Rev..

[38] Schmid P., Rauber D., Betsch C., Lidolt G., Denker M.L. (2017). Barriers of Influenza Vaccination Intention and Behavior-A Systematic Review of Influenza Vaccine Hesitancy, 2005–2016. PLoS ONE.

[39] Zhu Z., Guo M., Petrovsky D.V., Dong T., Hu Y., Wu B. (2019). Age and regional disparity in HIV education among migrants in China: Migrants population dynamic monitoring survey, 2014-2015. Int. J. Equity Health.

[40] Nie P., Ma W., Sousa-Poza A. (2021). The relationship between smartphone use and subjective well-being in rural China. Electron. Commer. Res..

[41] Olson J.A., Sandra D.A., Colucci É.S., Al Bikaii A., Chmoulevitch D., Nahas J., Veissière S.P. (2022). Smartphone addiction is increasing across the world: A meta-analysis of 24 countries. Comput. Hum. Behav..

[42] Wang Z., Wu Q., Ming J. (2020). The Relationship Between Homeownership and the Utilization of Local Public Health Services Among Rural Migrants in China: A Nationwide Cross-Sectional Study. Front. Public Health..

[43] Tyner A., Ren Y. (2016). The hukou system, rural institutions, and migrant integration In China. J. East. Asian Stud..

[44] Pickett K.E., Wilkinson R.G. (2015). Income inequality and health: A causal review. Soc. Sci. Med..

[45] Aalto A.M., Uutela A., Kangas T. (1996). Health behaviour, social integration, perceived health and dysfunction. A comparison between patients with type I and II diabetes and controls. Scand. J. Soc. Med..

[46] Larson H.J., Clarke R.M., Jarrett C., Eckersberger E., Levine Z., Schulz W.S., Paterson P. (2018). Measuring trust in vaccination: A systematic review. Hum. Vaccines Immunother..

[47] Leng A., Maitland E., Wang S., Nicholas S., Liu R., Wang J. (2021). Individual preferences for COVID-19 vaccination in China. Vaccine.

[48] Bruni L., Diaz M., Barrionuevo-Rosas L., Herrero R., Bray F., Bosch F.X., de Sanjosé S., Castellsagué X. (2016). Global estimates of human papillomavirus vaccination coverage by region and income level: A pooled analysis. Lancet Glob. Health.

[49] Fellmeth G., Rose-Clarke K., Zhao C., Busert L.K., Zheng Y., Massazza A., Sonmez H., Eder B., Blewitt A., Lertgrai W. (2018). Health impacts of parental migration on left-behind children and adolescents: A systematic review and meta-analysis. Lancet.

[50] Dubé E., Laberge C., Guay M., Bramadat P., Roy R., Bettinger J. (2013). Vaccine hesitancy: An overview. Hum. Vaccines Immunother..

[51] Luyten J., Beutels P. (2016). The Social Value of Vaccination Programs: Beyond Cost-Effectiveness. Health Aff..

[52] Omoniyi O.S., Williams I. (2020). Realist Synthesis of the International Theory and Evidence on Strategies to Improve Childhood Vaccination in Low- and Middle-Income Countries: Developing Strategies for the Nigerian Healthcare System. Int. J. Health Policy Manag..

[53] Cherian T., Morales K.F., Mantel C., Lambach P., Independent Expert Advisory Group (IEAG) for Health Worker Influenza Vaccination (2019). Factors and considerations for establishing and improving seasonal influenza vaccination of health workers: Report from a WHO meeting, 16–17 January, Berlin, Germany. Vaccine.

